# Supplementary figures and images for: Occupational bladder cancer: A cross section survey of previous employments, tasks and exposures matched to cancer phenotypes
Source: PLoS One. 2020 Oct 21;15(10):e0239338. doi: 10.1371/journal.pone.0239338 (PMC7577448; doi:10.1371/journal.pone.0239338)

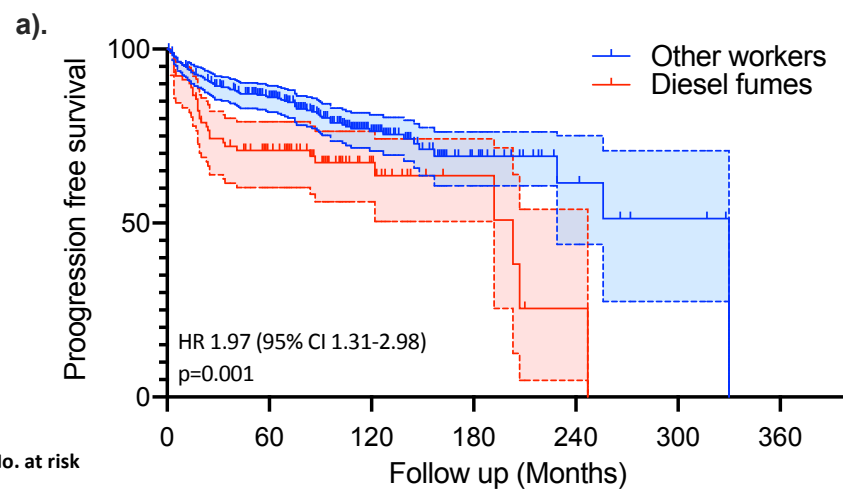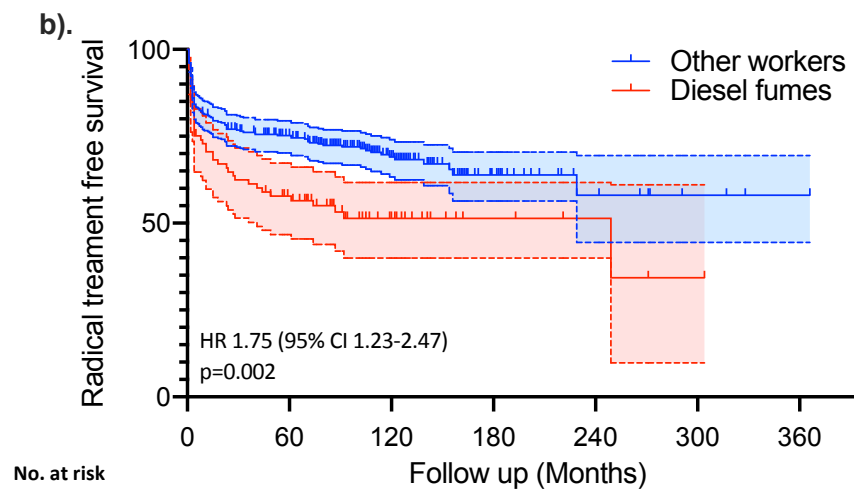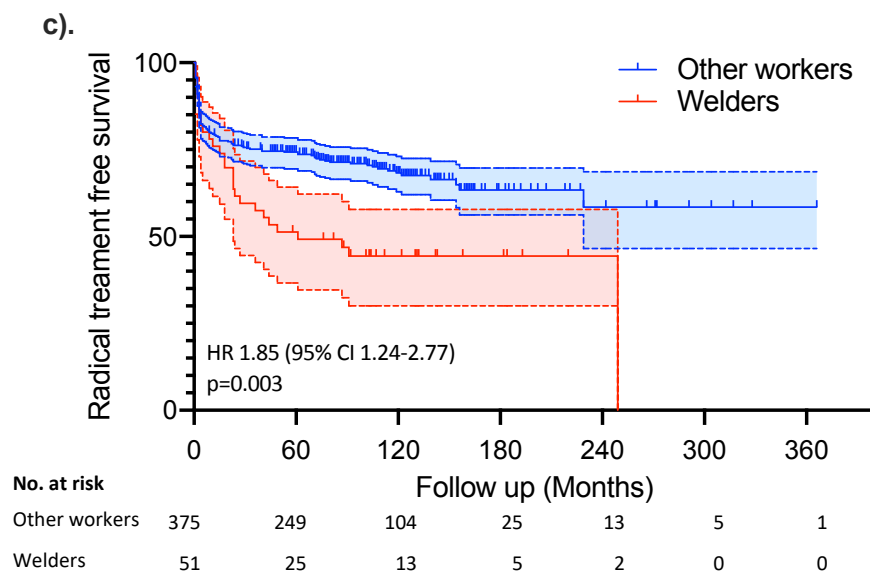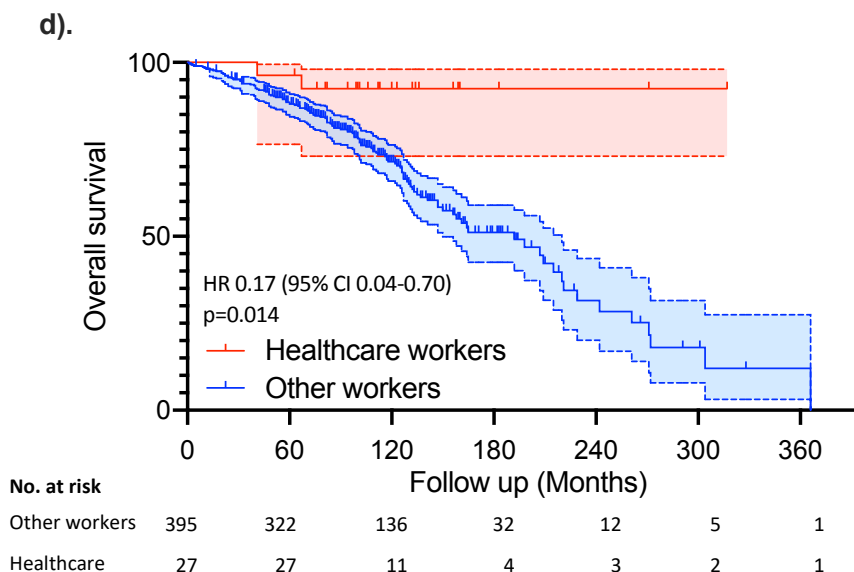

Supplement: S1 Fig — (PDF) [file pone.0239338.s001.pdf]
