# Supplementary material for: Occupational bladder cancer: A cross section survey of previous employments, tasks and exposures matched to cancer phenotypes
Source: PLoS One. 2020 Oct 21;15(10):e0239338. doi: 10.1371/journal.pone.0239338 (PMC7577448; doi:10.1371/journal.pone.0239338)
Supplement: S1 File — (PDF) [file pone.0239338.s002.pdf]

IMUC Study

Investigation into the Molecular Mechanisms of Urothelial Cancer

Participant Questionnaire Front Sheet

Please complete your name and date of birth on this sheet only.
Once your questionnaire is received by us we will remove this sheet so that your questionnaire becomes anonymous to all but Senior Staff in the Research Team.

Thank you very much.

1.01 **Today's Date: ……………………………………………………………….**

1.02 **Name:…………………………………………………………………..**

1.03 **DoB:…………………………………………………………………….**

1.04 **Post Code: ……………………………………………………………..**

1.05

NB: Please write in the box above how many minutes this questionnaire took you to complete.

Questionnaire for Urothelial Cancer Research

We are trying to identify some of the causes for urothelial cancer. We are asking people with and without this cancer to complete this questionnaire. We would like you to tell us about your job(s) in the past, and any substances that you may have been exposed to. We also ask about any history of cancer in your family.

Please answer the questions, even if you worked for as little as 6 months in the job or with the substance described.

On the next page, please complete your occupational history for any jobs where you have dealt with or been exposed to any types of chemical or fumes.

We use this information to build a picture of peoples' exposure to chemicals in relation to diseases they develop. We may contact you if there is a possibility that you are eligible for compensation for work related illness.

Year from	Employer **(or at**	Job Title	Tasks involved in this	Describe

and Year to **(very approximate is ok)** - from first job onwards	**least type of place of work** eg. steel factory)		job	approximately how often you carried out this task in this job (for example, every day, most days, once a week, once a month, once or twice a year)
1 2.00	2.01	2.02	2.03	2.04
2 2.05	2.06	2.07	2.08	2.09
3 2.10	2.11	2.12	2.13	2.14
4 2.15	2.16	2.17	2.18	2.19
5 2.20	2.21	2.22	2.23	2.24
6 2.25	2.26	2.27	2.28	2.29

1. **Employment Details -**

Year From and Year To **(very approximate is ok)** - from first job onwards	Employer **(or at least type of place of work** e.g. steel factory)	Job Title	Tasks involved in this job	Describe approximately how often you carried out this task in this job (for example, every day, most days, once a week, once a month, once or twice a year)
7 2.30	2.31	2.32	2.33	2.34
8 2.35	2.36	2.37	2.38	2.39
9 2.40	2.41	2.42	2.43	2.44
10 2.45	2.46	2.47	2.48	2.49
11 2.50	2.51	2.52	2.53	2.54
12 2.55	2.56	2.57	2.58	2.59

Please tick whichever substances you have ever worked with, even if you have worked with them for only a few months.

		Please tick
3.00	Dyes	
3.01	Crack-detection dyes	
3.02	Dyeing material	
3.03	Any other type of dye or stain	
3.04	Cadmium	
3.05	Chromium	
3.06	Coal, gas and oil by product chemicals	
3.07	Gas works sludge	
3.08	Coking plant fumes or residues	
3.09	Coal or coal products	
3.10	Cooking fumes	
3.11	Diesel exhaust fumes	
3.12	Oily/greasy rust proofing chemicals	
3.13	Diesel fuel	
3.14	Aircraft fuel	
3.15	DDM or MDA	
3.16	MOCA	
3.17	Printers' ink	
3.18	Solvents	
	e.g. trichloroethylene ('trike') perchloroethylene	
	anything with 'chlor*' in the name	
3.19	Arsenic	
3.20	Fungicide, wood preservative (e.g. Nitrobiphenyl).	
3.21	o-toluidine	
3.22	4-aminobiphenyl	
3.23	Ionising radiation (radioactive sources)	
3.24	Coal tar cream	

This page may seem similar to the first page of the questionnaire but we would be grateful if you could complete it.
Please tick any types of industry below that you have worked in, even if you were only there for a short period of time (e.g, 6 months) and even if it was a long time ago.

	Type of Industry		Please tick	Job title
4.00	Coke, coal, power generation	Coking plant or gas production		
4.01		Coal mining or smokeless fuel making		
4.02		Nuclear power		
4.03	Steel and foundry	Metal refining		
4.04		Steel industry		
4.05		Steel production		
4.06		Heat treatment		
4.07		Forging		
4.08		Foundries		
4.09	Engineering and metals	Engineering,		
4.10		Electroplating		
4.11		Cutlery		
4.12		Welding		
4.13		Making electrical contacts and solder		
4.14		Soldering		
4.15	Other manufacturing	Refining, and recycling (including electrical goods, light bulbs, TV tubes)		
4.16		Making garments and textiles		
4.17		Spinning synthetic fibre		
4.18		Plastic production		
4.19		Cement products		
4.20		Rubber industry and tyre industry		
4.21		Chemical industry		
4.22		Petroleum industry		
4.23	Services	Laundries		
4.24		Hairdressing		
4.25		Health care		
4.26	Farming, gardening	Agriculture,		
4.27		Horticulture		
4.28	Building trade	Construction		
4.29		Painting		
4.30	Transport and related	Garages		
4.31		Nuclear power		
4.32		Driving jobs		
4.33		Warehousing		
4.34		Engine repairs		

Have you ever carried out any of the following tasks, or worked nearby to someone who was carrying out one of these tasks?

	Task	In my own job	Done nearby me at work	
5.00	Smelting metals			5.01
5.02	Assembling and repairing electrical goods			5.03
5.04	Making products containing cadmium			5.05
5.06	Making or using cement			5.07
5.08	Soldering or brazing			5.09
5.10	Metal plating			5.11
5.12	Cadmium plating			5.13
5.14	Fumes from quenching (heat treatment)			5.15
5.16	Fumes from forging			5.17
5.18	Crack detection /Non-destructive testing			5.19
5.20	Resins in ‘cold box’ techniques in foundries			5.21
5.22	Contact with weld material and steel			5.23
5.24	Fume from producing and using coke, and converting coal to gas.			5.25
5.26	Residues from coke and gas production			5.27
5.28	Making or handling plastics			5.29
5.30	Making or handling rubber products			5.31
5.32	Breakdown of resins used to make moulds and cores			5.33
5.34	Making chemicals from coal, coke, oil and gas byproducts			5.35
5.36	e.g. additives to aeroplane fuel			5.37
5.38	Mineral oils used as lubricants and coolants			5.39
5.40	Making and using resins: coatings for surfaces of metal objects e.g corrosion resistance, rust-proofing			5.41
5.42	Making plastic foam			5.43
5.44	Degreasing			5.45
5.46	Dry-cleaning			5.47
5.48	Timber treatment			5.49
5.50	Plumbing, gas-fitting, heat and ventilation fitting			5.51
5.52	Painting e.g. metal coatings<br>Dyeing material – paper, textiles, anything (lacquers)			5.53
5.54	Contact with industrial diesel			5.55
5.56	Separated out impurities, ores, scrap or wastes			5.57
5.58	Pesticide and herbicide treatments			5.59
5.60	Burning plastics			5.61
5.62	Radiotherapy			
5.64	Industrial radiography			

If you have had exposure to a particular chemical, please could you write this chemical down and then describe the contact that you had with it.

	Chemical	<u>Type of Contact</u> - please consider these points when describing contact.... • Skin contact/breathing in/tasting it • How long for? How many days a week? For how many months/years? • Did you wear a mask or gloves when handling this chemical?
6.01	1	
6.02	2	
6.03	3	
6.04	4	
6.05	5	
6.06	6	
6.07	7	
6.08	8	

6.09

If you have not yet stated on this questionnaire what your occupation has been over the years, please could you write it here:
..

..

Thank you.

About other activities (please circle 'yes' or 'no')

**Smoking history**		
Are you **or** have you ever been a smoker ?	Yes / No 7.00	
How old were you when you started ?	7.02	
How much do you smoke a day if you smoke now?	Cigarettes 7.03	Tobacco (ounces) 7.04
If you stopped, how old were you?	7.05	
How much did you smoke per day ?	Cigarettes 7.06	Tobacco (ounces) 7.07
**Passive smoking history**		
Have you ever lived with someone who smoked in the house ?	Yes / No 7.09	Date (or age) from: 7.10
		Date (or age) to: 7.11
Have you ever worked in an environment in which people smoked ?	Yes / No 7.13	Date (or age) from: 7.14
		Date (or age) to: 7.15
**Have you had any of these hobbies?**		
Fishing	Yes / No 7.17	If so, for how 7.18 many years ?
Swimming	Yes / No 7.20	If so, for how 7.21 many years ?
Model building, e.g. train/car/aeroplane/	Yes / No 7.23	If so, for how 7.24 many years ?
**Have you used hair dyes ?**	Yes / No 7.26	
Which dye products?	7.27	
Which colours regularly ?	7.28	
**Have you used any of the following medications ?**		
Phenacetin	Yes / No 7.30	
Coal tar creams	Yes / No 7.32	
**Have you lived in any area of the country other than South Yorkshire ?**	Yes / No 7.34	
Where ?	For how long ? 7.35	
Where ?	For how long ? 7.36	
**If you currently have or have had a growth in your bladder, ureter or kidney, do you have any thoughts on what may have contributed to you developing this?**	Comments: 7.37	

8. Family Cancer History

Please could you note down below any of your blood relatives who have had any type of cancer, how old they were (approximately age if not known) and what type of cancer they had, plus any further comments you wish to make. **If you have no family cancers to note then please write 'none known'.** Thank you.

Relative	Type of cancer	Age diagnosed	Comments
8.00	8.01	8.02	8.03
8.04	8.05	8.06	8.07
8.08	8.09	8.10	8.11
8.12	8.13	8.14	8.15
8.16	8.17	8.18	8.19
8.20	8.21	8.22	8.23

Have you ever been diagnosed with any type of cancer in the past ?8.50

yes / no

Please state what type of cancer: 8.51……………………………………………

Approximately which year was this cancer diagnosed ? 8.52…………………
