# Supplementary material for: Occupational bladder cancer: A cross section survey of previous employments, tasks and exposures matched to cancer phenotypes
Source: PLoS One. 2020 Oct 21;15(10):e0239338. doi: 10.1371/journal.pone.0239338 (PMC7577448; doi:10.1371/journal.pone.0239338)
Supplement: Attachment — Submitted filename: PLOS one occupational.docx [file pone.0239338.s003.docx]

Reviewer 1 : minor revisions

The submitted manuscript “Occupational Bladder cancer: A cross section survey of previous employments, tasks and exposures matched to cancer phenotypes.” by Reed et al. reports on 454 patients with urothelial bladder cancer, first diagnosed and treated between 02/2010 - 07/2012 at a single institution, the RHH (Sheffield).

Based on a patient-reported questionnaire, patients were evaluated for potential carcinogen exposure, whilst occupational classes were assigned using NYK and ISCO-1958 codes.

With a median follow-up of 8.4 years, the authors additionally report on tumor progression and the need of radical intervention. Outcome data was collected between 08/2018 - 10/2018.

This questionnaire-based evaluation, revealed multiple occupational tasks and contacts associated with high grade and high stage BC. Tumors were classified according to TNM and WHO 1973 criteria, therefore G1-G3 grading data has been reported.

Typical for an urothelial cancer population is the distribution between men and women with a ratio of roughly 4:1. Therefore, the reported data refers to mainly men, since all patients were included at time of initial diagnosis of bladder cancer.

The authors found differences in exposures between men and women, including

distinct patterns of employment, differences in smoking rates and patterns,

hair dye use and hobbies.

When only female patients were analyzed, the data suggests an association between high grade/high stage BC and workers undertaking electroplating and cutlery manufacture, and tasks such as degreasing and painting.

Although, the included patient cohort quite small to evaluate potential influences of exposure to occupational carcinogens, there are some interesting findings, worth to be reported.

Limitations of the manuscript are well described (e.g. estimated duration of each task), data reported and analyzed with appropriate methods, and outcome data revealed quite distinct differences for specific occupational groups.

Overall, an interesting and well-written manuscript, worth publication after minor revision:

Some comments:

-	Please use BOLD for all significant values, which makes it easier to read tables.

-	For Kaplan-Meier curves, please add “at risk numbers” on the bottom of the graphs.

Reviewer 2 : major revisions

Paper Occupational bladder cancer: A cross section survey of previous employments, tasks and exposures matched to cancer phenotypes by dr. Reed et al.
Very interesting study. I have just few comments, which, as I believe, can improve paper.
1, I would stress time of exposure. I believe it is very important point missed in discussion and even in abstract
2, I would suggest also hobby of participants. It is well known from other epidemiological studies......example can be psittacosis. It is indeed mostly occupational exposure linked disease, however substantial part of patients came from hobby sector (bird breeders, parrot lovers, etc, etc). Authors listed garage work as potential risk factor for aggressive disease course with recquired radical treatment. There are many car lovers, bikers who spend a substantial time in care of their gear and indeed they are haevily exposed to risk substanties. I think this should be at least discussed.
3, Is there any chance to check any link between variant histology (mostly highly aggressive tumors, sometimes with bland cytology-ie LG, like nested UC) and occupational exposure
