# Supplementary material for: Occupational bladder cancer: A cross section survey of previous employments, tasks and exposures matched to cancer phenotypes
Source: PLoS One. 2020 Oct 21;15(10):e0239338. doi: 10.1371/journal.pone.0239338 (PMC7577448; doi:10.1371/journal.pone.0239338)
Supplement: Attachment — Submitted filename: Response to Reviewers.docx [file pone.0239338.s004.docx]

| 10 June 2020  Pr. Eva Compérat Academic Editor PLOS ONE |  | Professor C Newman, Pro VC for medicine  **Department of Oncology and Metabolism,**  James Catto MB ChB, PhD, FRCS(Urol), FRSB  NIHR Research Professor  and Professor of Urological Surgery  Academic Urology Unit, University of Sheffield,  The Medical School, G Floor,  Beech Hill Road, Sheffield S10 2JF,  United Kingdom  Office Telephone: +44 (0)114 271 2163  PA Telephone: +44 (0)114 226 1229  Fax: +44 (0)114 271 2268  Email: J.Catto@sheffield.ac.uk |
| --- | --- | --- |

Dear Professor Compérat,

**Re: PONE-D-20-10549 Occupational Bladder cancer: A cross section survey of previous employments, tasks, exposures matched to cancer phenotypes**

Thank you for the helpful comments and time spent looking at our work. I hope you are safe and well in these difficult times.

We have revised the manuscript in keeping with the reviewer’s comments. We would like to thank you for your review of the manuscript and we feel it has been improved by the comments. Below we have outlined responses to each point.

Yours sincerely

Best wishes and Yours Sincerely

James Catto

Reviewer(s)' Comments to Author:

**Editorial comments:**

1. Please ensure that your manuscript meets PLOS ONE's style requirements, including those for file naming. The PLOS ONE style templates can be found at

<https://journals.plos.org/plosone/s/file?id=wjVg/PLOSOne_formatting_sample_main_body.pdf> and

<https://journals.plos.org/plosone/s/file?id=ba62/PLOSOne_formatting_sample_title_authors_affiliations.pdf>

**Answer:** Thank you, this has been amended and actioned.

2. Thank you for including your ethics statement:

'All patients underwent informed consent in an ethically approved programme (South Yorkshire Research Ethics Committee approval number 10/H1310/73) before entry.'

a. Please amend your current ethics statement to confirm that your named institutional review board or ethics committee specifically approved this study.

b. Once you have amended this/these statement(s) in the Methods section of the manuscript, please add the same text to the “Ethics Statement” field of the submission form (via “Edit Submission”).

For additional information about PLOS ONE ethical requirements for human subjects research, please refer to <http://journals.plos.org/plosone/s/submission-guidelines#loc-human-subjects-research>

**Answer:** Yes, this was specifically approved by Sheffield Teaching Hospitals review board. Ethics Statement has been amended and I will add this to the Submission form on re-submission.

3. Please include additional information regarding the survey or questionnaire used in the study and ensure that you have provided sufficient details that others could replicate the analyses.

For instance, if you developed a questionnaire as part of this study and it is not under a copyright more restrictive than CC-BY, please include a copy, in both the original language and English, as Supporting Information.

**Answer:** PDF of Questionnaire is provided as a supplementary file.

4. Please provide a sample size and power calculation in the Methods, or discuss the reasons for not performing one before study initiation.

**Answer:** No formal power calculation was performed. This study was an explorative cohort study rather than a clinical trial. As such, we included all eligible patients in the recruiting time frame and aimed to describe data to allow future studies to be powered accordingly. We have added this sentence in the discussion: *“Our aim was to undertake an explorative cohort study (rather than a clinical trial) and so no formal power calculation was performed. This reflects that very little is known about occupational risks and bladder cancer phenotypes and so powering was not possible. Our findings …..”*

5. Your ethics statement must appear in the Methods section of your manuscript. If your ethics statement is written in any section besides the Methods, please move it to the Methods section and delete it from any other section. Please also ensure that your ethics statement is included in your manuscript, as the ethics section of your online submission will not be published alongside your manuscript.

**Answer**: Thank you, this has been amended.

6. Please include a caption for figure 1.

**Answer:** Thank you, this has been amended and actioned.

7. Please include captions for your Supporting Information files at the end of your manuscript, and update any in-text citations to match accordingly. Please see our Supporting Information guidelines for more information: <http://journals.plos.org/plosone/s/supporting-information>

**Answer:** Captions and in-text citations matched accordingly – I have subcategorised them as Fig. 1 a-d. Please see if you are happy with this.

**Reviewer: 1**

Overall, an interesting and well-written manuscript, worth publication after minor revision:

Some comments:

-	Please use BOLD for all significant values, which makes it easier to read tables.

**Answer:** Thank you, this has been amended and actioned.

-	For **Kaplan-Meier curves, please add “at risk numbers” on the bottom of the graphs.**

**Answer:** Thank you this has been amended and actioned

**Reviewer 2 : major revisions**

Paper Occupational bladder cancer: A cross section survey of previous employments, tasks and exposures matched to cancer phenotypes by dr. Reed et al. Very interesting study. I have just few comments, which, as I believe, can improve paper.

1, I would stress time of exposure. I believe it is very important point missed in discussion and even in abstract

**Answer:** Thank you, this has been amended and actioned.

2, I would suggest also hobby of participants. It is well known from other epidemiological studies......example can be psittacosis. It is indeed mostly occupational exposure linked disease, however substantial part of patients came from hobby sector (bird breeders, parrot lovers, etc, etc). Authors listed garage work as potential risk factor for aggressive disease course with required radical treatment. There are many car lovers, bikers who spend a substantial time in care of their gear and indeed they are heavily exposed to risk substances. I think this should be at least discussed.

**Answer:** Thank you. As can be seen in the questionnaire (added as supplementary questions), we did ask about bladder cancer related hobbies. We have not included this data as 1. Many replies were empty, … 2. So there were too few to be meaningful for analysis, and 3. The manuscript already has a lot of information analyses/data/tables and so any added messages would be too unclear.

3, Is there any chance to check any link between variant histology (mostly highly aggressive tumors, sometimes with bland cytology-ie LG, like nested UC) and occupational exposure

**Answer:** This is a very valuable point. Indeed some cases were defined as variants in their pathological reports. However, in 2010, the presence or absence of variant histology was not reliably reported in our hospital or in the UK (please see Urol Oncol. 2013 Nov;31(8):1650-5). As such, we are unable to reliably know whether each case had/did not have variant patterns and so have not reported this.
